# High-throughput Screening of Small Molecule Inhibitors of the *Streptococcus* Quorum-sensing Signal Pathway

**DOI:** 10.1038/s41598-017-03567-2

**Published:** 2017-06-22

**Authors:** Seiji Ishii, Kenji Fukui, Satoshi Yokoshima, Kazuo Kumagai, Youko Beniyama, Tetsuya Kodama, Tohru Fukuyama, Takayoshi Okabe, Tetsuo Nagano, Hirotatsu Kojima, Takato Yano

**Affiliations:** 1 Department of Biochemistry, Osaka Medical College, Takatsuki, Osaka 569-8686 Japan; 2 Graduate School of Pharmaceutical Sciences, Nagoya University, Furo-cho, Chikusa-ku, Nagoya 464-8601 Japan; 3 Drug Discovery Initiative, The University of Tokyo, 7-3-1 Hongo, Bunkyo-ku, Tokyo 113-0033 Japan; 4 Center for Membrane and Film Technology, Graduate School of Engineering, Kobe University, Kobe, Japan

## Abstract

The main components of the quorum-sensing system are expected to be favorable targets for drug development to combat various chronic infectious diseases. ComA of *Streptococcus* is an ATP-binding cassette transporter containing a peptidase domain (PEP), which is essential for the quorum-sensing signal production. Using high-throughput screening, we found a potent small molecule that suppressed the *S. mutans* quorum-sensing pathway through inhibition of PEP activity. The compound effectively attenuated the biofilm formation and competence development of *S. mutans* without inhibiting cell growth. The kinetic and structural studies with this molecule and a related compound unexpectedly revealed an allosteric site of PEP. This relatively hydrophobic site is thought to undergo large structural changes during the catalytic process. These compounds inhibit PEP activity by binding to and suppressing the structural changes of this site. These results showed that PEP is a good target for inhibitors of the *Streptococcus* quorum-sensing system.

## Introduction

*Streptococcus* is a genus of Gram-positive bacteria that consists of a wide variety of pathogenic and commensal species. Some commensal species are known to be opportunistic pathogens. Notably, oral streptococci, such as *S. mutans*, are not only cariogenic but also occasionally enter the human circulatory system and cause life-threatening infective endocarditis by forming a biofilm on the native or prosthetic heart valves, especially in patients from Asia^1, 2^. Formation of the biofilm, together with its inherent resistance to antibiotics, is the main factor in the chronic and refractory nature of this infection^3^. Generally, bacterial biofilm formation is thought to be regulated by the quorum-sensing system^4^.

The quorum-sensing system is a bacterial cell–cell signal communication system mediated by an inherent signal molecule called an autoinducer^5^. The ComABCDE pathway is the quorum-sensing system in some species of *Streptococcus*, such as *S. mutans* and *S. pneumoniae*, in which autoinducer peptides are processed from the precursor ComC and concomitantly exported to the extracellular space by ComA and ComB^6, 7^. The accumulated autoinducers bind to the membrane-bound receptor kinase ComD, which subsequently phosphorylates the response regulator ComE to activate transcription of a specific set of genes, such as those essential for the competence development in *S. mutans* ^6^, *S. pneumoniae* ^7, 8^, and *S. gordonii* ^9^ and the biofilm formation in *S. mutans* ^10^ and *S. pneumoniae* ^11^. It is expected that inhibitor development against this system would provide a way to design drugs for various clinical conditions caused by chronic biofilm infections. One purported benefit of quorum-sensing inhibitors is that, because they do not directly kill bacterial cells, they should exert lower selection pressure and, hence, be less susceptible to development of drug resistance than are antimicrobials^12^.

**Figure 1:**
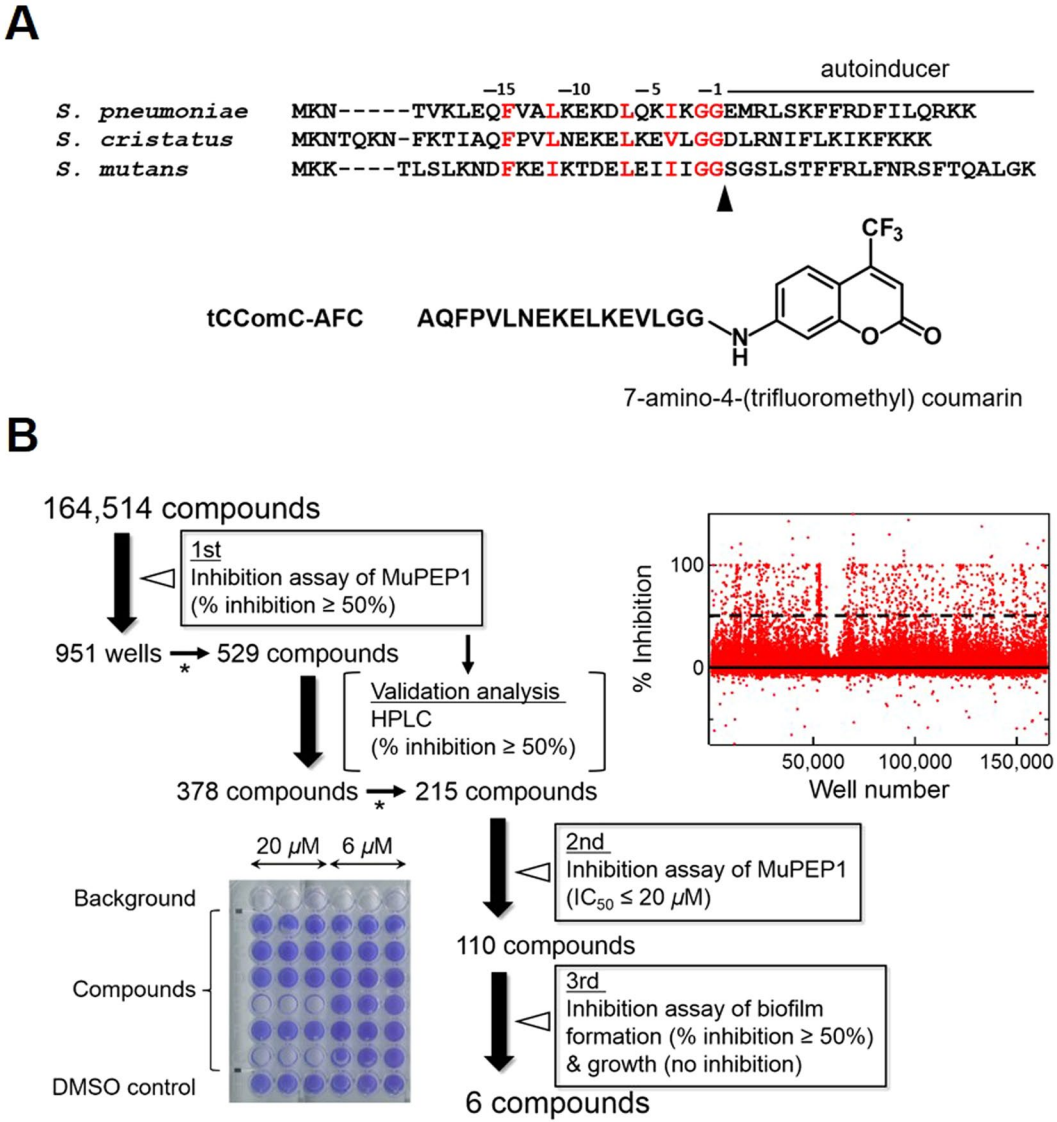
High-throughput Screening Using a Fluorescence-labeled Substrate to Identify Effective MuPEP1 Inhibitors. (**A**) Sequence alignment of ComCs from *S. pneumoniae*, *S. cristatus*, and *S. mutans* and the structure of tCComC-AFC. Highly conserved hydrophobic amino acid residues and the consensus Gly–Gly are colored red. The arrowhead indicates the cleavage site of ComC by PEP. (**B**) Flow chart of high-throughput screening to identify compounds that inhibit the MuPEP1 activity and the biofilm formation of *S. mutans*. Results of the first screening are shown (upper right). The broken line indicates 50% inhibition. An example of the results from the third screening (in triplicate at two different compound concentrations) is also shown (lower left). The asterisks show that hit compounds were narrowed down by visual inspection of compound structures.

ComA is a bi-functional ATP-binding cassette (ABC) transporter that comprises three domains: an N-terminal peptidase domain (PEP), a transmembrane domain, and a C-terminal nucleotide-binding domain^13–15^. PEP is a highly specific peptidase belonging to a cysteine protease family^13, 16–18^. We have previously elucidated the substrate recognition mechanism of PEP^17, 18^ in which the tight cleft at the active site of PEP binds the Gly–Gly motif of ComC and a shallow hydrophobic concave surface of PEP accommodates the conserved hydrophobic residues in the N-terminal *α*-helical region of ComC (Fig. 1A).

Understandably, development of inhibitors for the quorum-sensing system of Gram-positive bacteria has mainly targeted the signal–receptor interaction, that is, substrate-mimetic receptor inhibitors^19, 20^. One major drawback of this strategy is that amino acid sequences of autoinducer peptides are highly variable among bacterial species, or in some cases even among strains, and therefore, labor-intensive drug development would have to be done for each species. We believe that PEP of ComA is a more suitable target for inhibitor development for the following reasons: First, PEP catalyzes the initial step of the quorum-sensing system of *Streptococcus*. Second, among ubiquitous ABC transporters, ComA-like transporters equipped with the peptidase domains are found only in prokaryotes, thus minimizing the possibility of unpredictable adverse effects^13^. Third, because, as far as we have been able to determine, all streptococcal PEPs have a common substrate recognition mechanism^17^, it may be possible to develop a quorum-sensing inhibitor that is effective for a range of streptococcal species in which PEPs play an important role in the quorum-sensing system.

In this study, we set out to screen a library of small compounds for the inhibitory activity against *S. mutans* PEP (MuPEP1) by high-throughput screening.

## Results and Discussion

### High-throughput Screening of PEP Inhibitors

We established a high-throughput screening system using a fluorescence-labeled substrate (tCComC-AFC) (Fig. 1B and Supplementary Fig. S1). *S. cristatus* ComC (CComC) was used because *S. mutans* ComC could not be chemically synthesized and CComC was a good substrate of MuPEP1^17^. The first screening of 164,514 compounds (Z′-factor value of 0.93, Supplementary Fig. S2) yielded 951 hits (0.58%) that inhibited MuPEP1 activity by >50% at a compound concentration of 20 *μ*M. After the inhibitory activities of the selected compounds were re-evaluated by high-performance liquid chromatography assay, dose-dependent inhibition against MuPEP1 was examined in a second screening that yielded 110 compounds with IC_50_ values of <20 *μ*M. To check both the cell permeability and *in vivo* activity, these compounds were further subjected to a third screening that examined inhibition against *S. mutans* biofilm formation. Some compounds inhibited biofilm formation but also showed antimicrobial toxicity to *S. mutans*, which indicated some non-specific *in vivo* activities. Finally, six compounds were found to inhibit biofilm formation without inhibiting cell growth. Two of the six compounds were quinuclidine derivatives, and the other four compounds had no primary chemical structure in common.

### Effects of Compound 1 on the Quorum-sensing Pathway

**Figure 2:**
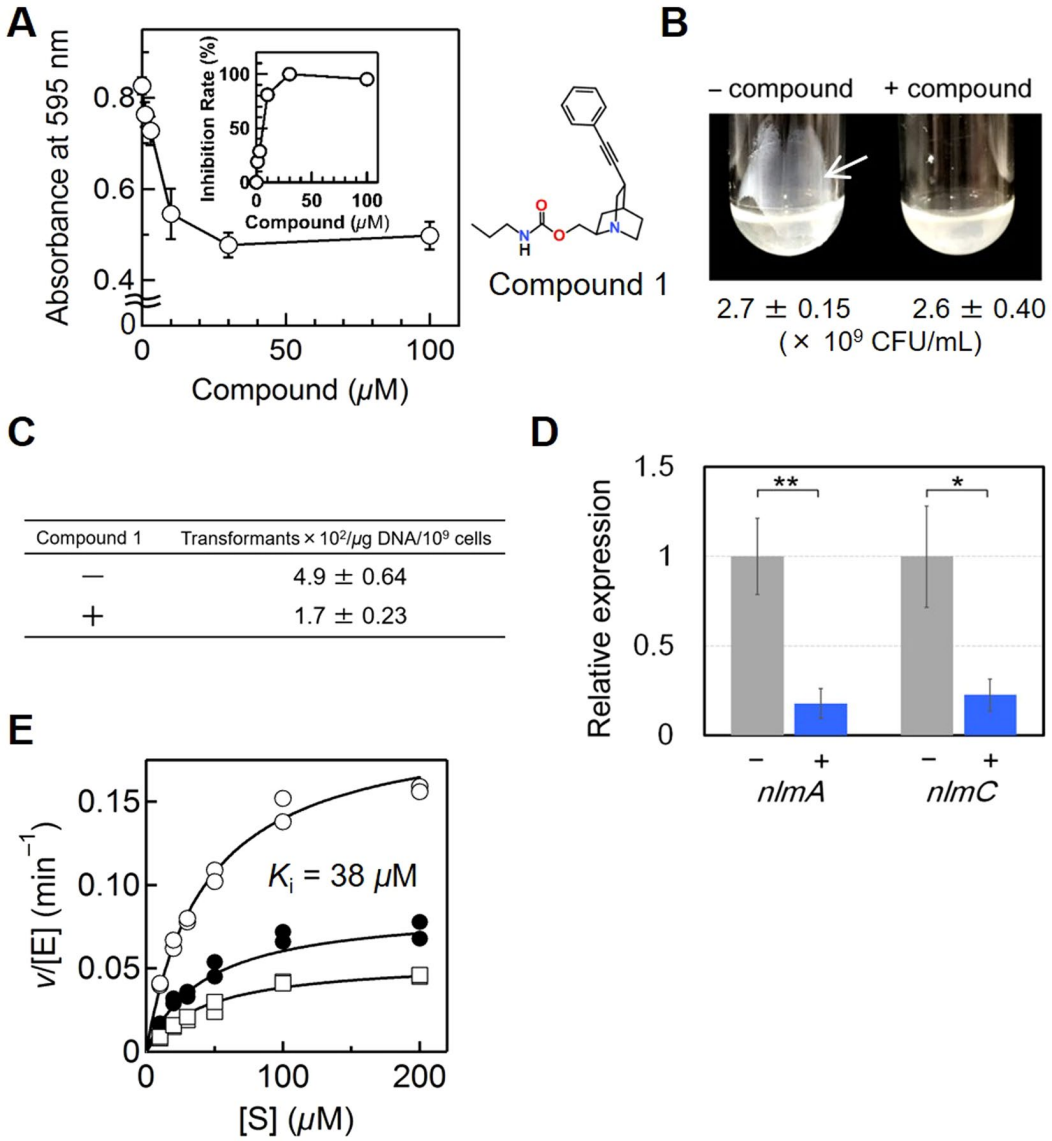
Examination of *in vivo* and *in vitro* Activities of Compound 1. (**A**) Biofilm mass of *S. mutans* (means ± SD, n = 3) in the presence of various concentrations of Compound 1 estimated by crystal violet staining. Inhibition rates were calculated with the maximum suppression of biofilm formation as 100% (*inset*). (**B**) Images of the biofilm of *S. mutans* in the absence or presence (25 *μ*M) of Compound 1 formed on the walls of polystyrene test tubes. The white arrow indicates the biofilm of *S. mutans*, which formed on the wall of a tilted tube after the overnight standing culture. Numbers of viable cells from the same experiment (means ± SD, n = 3) are indicated below (CFU, colony forming unit). (**C**) Transformation efficiency (means ± SD, n = 3) of *S. mutans* in the absence or presence (10 *µ*M) of Compound 1. (**D**) Relative mRNA expression levels (means ± SD, n = 3) of the *nlmA* and *nlmC* genes of *S. mutans* in the absence or presence (25 *μ*M) of Compound 1 were examined by quantitative RT-PCR (*p = 0.017, **p = 0.0045). (**E**) *v*/[E] versus substrate concentrations plots of the MuPEP1 peptidase assay for tCComC-AFC in the absence (○) or presence (●, 50 *μ*M and □, 100 *μ*M) of Compound 1 (n = 2). Theoretical curves were fitted by the non-competitive inhibition model with the parameters of *k*_cat_ = 0.20 min^−1^, *K*_m_ = 43 *μ*M, and *K*_i_ = 38 *μ*M.

To validate the outcome of the whole screening process, these compounds and some of their derivatives were (re-)synthesized or purchased, and their inhibitory activities against MuPEP1 were evaluated. We found one potent compound that showed an IC_50_ value of 38 *μ*M (Supplementary Fig. S3), and this compound with a quinuclidine core was designated Compound 1 (see Supplementary Methods for detailed information about the compound). We first examined the *S. mutans* biofilm formation in the presence of various concentrations of Compound 1 by using the same method as used in the third screening. The biofilm formation was dose-dependently suppressed by Compound 1, with an EC_50_ value of 5 *μ*M (Fig. 2A). A high background (value of approximately 0.5 for absorbance at 595 nm) of this method would be due to adherent bacteria on the flat bottom of the microtiter plate after overnight standing of the culture. The inhibitory effect was more clearly shown by the standing culture in tilted round-bottom culture tubes (Fig. 2B). Although the biofilm formed in the absence of Compound 1 remained tightly stuck to the wall of the tube after shaking, the biofilm formed in the presence of 25 *μ*M Compound 1 was easily detached and dispersed after gentle swirling. It is noteworthy that Compound 1 did not show antimicrobial activity against *S. mutans* under this condition (Fig. 2B). The effect of Compound 1 on the competence development of *S. mutans* was also evaluated. The transformation efficiency of *S. mutans* decreased to 35% in the presence of 10 *μ*M Compound 1 (Fig. 2C). To examine the effect of Compound 1 downstream of the quorum-sensing pathway, the expression levels of two bacteriocin genes, *nlmA* and *nlmC*, were estimated by quantitative RT-PCR. These genes are unrelated to biofilm formation or competence development but are well known to be directly regulated by the response regulator ComE^21^. As shown in Fig. 2D, the relative gene expression levels of *nlmA* and *nlmC* were suppressed to 18% and 23% by 25 *μ*M Compound 1, respectively. These results showed that Compound 1 was efficiently taken up into the cell, inhibited PEP activity, and effectively perturbed quorum-sensing signaling, which eventually should lead to poor biofilm formation and low transformation efficiency of *S. mutans*.

To assess the inhibition mechanism of Compound 1, kinetic analysis was performed at various concentrations of tCComC-AFC in the presence of 0, 50, and 100 *μ*M of Compound 1 (Fig. 2E, the Lineweaver–Burk plot in Supplementary Fig. S4). The data (V_max_ decreased, *K*_m_ was unaffected) demonstrated that Compound 1 non-competitively inhibited MuPEP1, with a *K*_i_ value of 38 *μ*M. From these results, we assumed that Compound 1 is an allosteric inhibitor, which binds to a site apart from the catalytic center of MuPEP1. To address this idea, we tried to resolve the structure of MuPEP1 complexed with Compound 1 by soaking and co-crystallization methods under various conditions, but the diffraction data revealed no bound compound molecule.

### Identification and Characterization of the Allosteric Site

**Figure 3:**
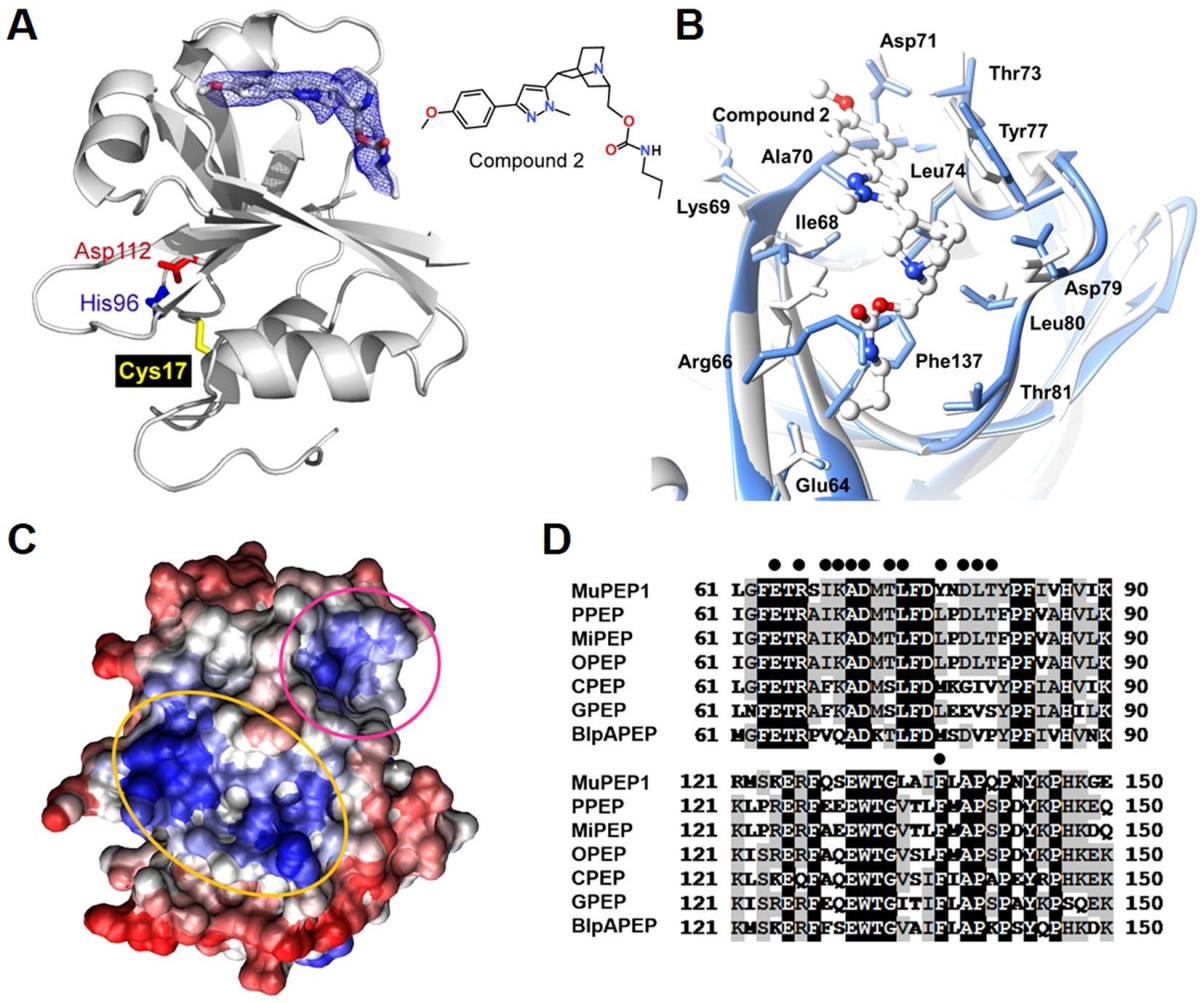
Structure of the MuPEP1–Compound 2 Complex and the Compound 2-binding Site. (**A**) The crystal structure of the tMuPEP1–Compound 2 complex and an |*F*_*o*_|−|*F*_*c*_| omit map. The electron density is shown around the compound and contoured at a level of 2.5 σ (blue mesh). The side chains of the catalytic triad residues, Cys17 (yellow), His96 (blue), and Asp112 (red), are shown as sticks. The oxygen and nitrogen of Compound 2 are colored red and blue, respectively. (**B**) Superimposition of the structures of the free tMuPEP1 (cyan) and the tMuPEP1-Compound 2 complex (white). The residues that interact with Compound 2 are shown as sticks and are labeled. The oxygen and nitrogen of Compound 2 are colored red and blue, respectively. (**C**) Surface representation of the result of the HotPatch program performed on MuPEP1^33^. The hydrophobic region is colored blue, and the hydrophilic region is red. The hydrophobic concave surface that is the binding site for the N-terminal helix of ComC is encircled by an orange line, and the inhibitor binding pocket is encircled by a magenta line. (**D**) Amino acid sequence alignment of PEPs from different species of *Streptococcus* ComA and the peptidase domain of *S. pneumoniae* BlpA. The completely identical amino acid residues and the partially identical amino acid residues (at least four out of seven sequences) among PEP sequences are shaded black and gray, respectively. MuPEP1, *S. mutans* PEP; PPEP, *S. pneumoniae* PEP; MiPEP, *S. mitis* PEP; OPEP, *S. oralis* PEP; CPEP, *S. cristatus* PEP; GPEP, *S. gordonii* PEP; and BlpAPEP, the peptidase domain of *S. pneumoniae* BlpA. Dots indicate 13 amino acid residues that are in close contact with Compound 2 in the structure of the tMuPEP1-Compound 2 complex.

Therefore, we prepared another form of MuPEP1 with the four N-terminal residues truncated (tMuPEP1), a form that was found to be crystallized in a different space group from that of MuPEP1. We also tested several synthetic analogs of Compound 1 and revealed the structure of tMuPEP1 in complex with Compound 2 (Fig. 3A) at a 3.1 Å resolution (Supplementary Table S1). The electron density of v-shaped Compound 2 was clearly observed in a pocket composed of *β*-strand (Phe63–Lys69), *α*-helix (Asp71–Tyr77), loop (Asn78–Pro83), Ala70, and the side chain of Phe137 (Fig. 3B). The binding is mainly mediated through hydrophobic and van der Waals interactions. A few hydrogen bonds might also contribute, but the interactions cannot be specified at this resolution. Although the physiological function of this relatively hydrophobic pocket (Fig. 3C) is unknown, the residues interacting with Compound 2 are conserved among *Streptococcus* PEPs (Fig. 3D, dots). In the crystal structure of the original MuPEP1, this pocket is occupied by residues from the adjacent protein molecule, which would explain why the complex structure could not be obtained. The side chain of Arg66 occupies the vacant space of this binding pocket in free tMuPEP1, whereas it is rotated away to avoid steric hindrance with Compound 2 in the tMuPEP1-Compound 2 complex (Fig. 3B). There are no other significant differences in either the overall structure or the catalytic triad residues between the free and complex structures of tMuPEP1.

**Figure 4:**
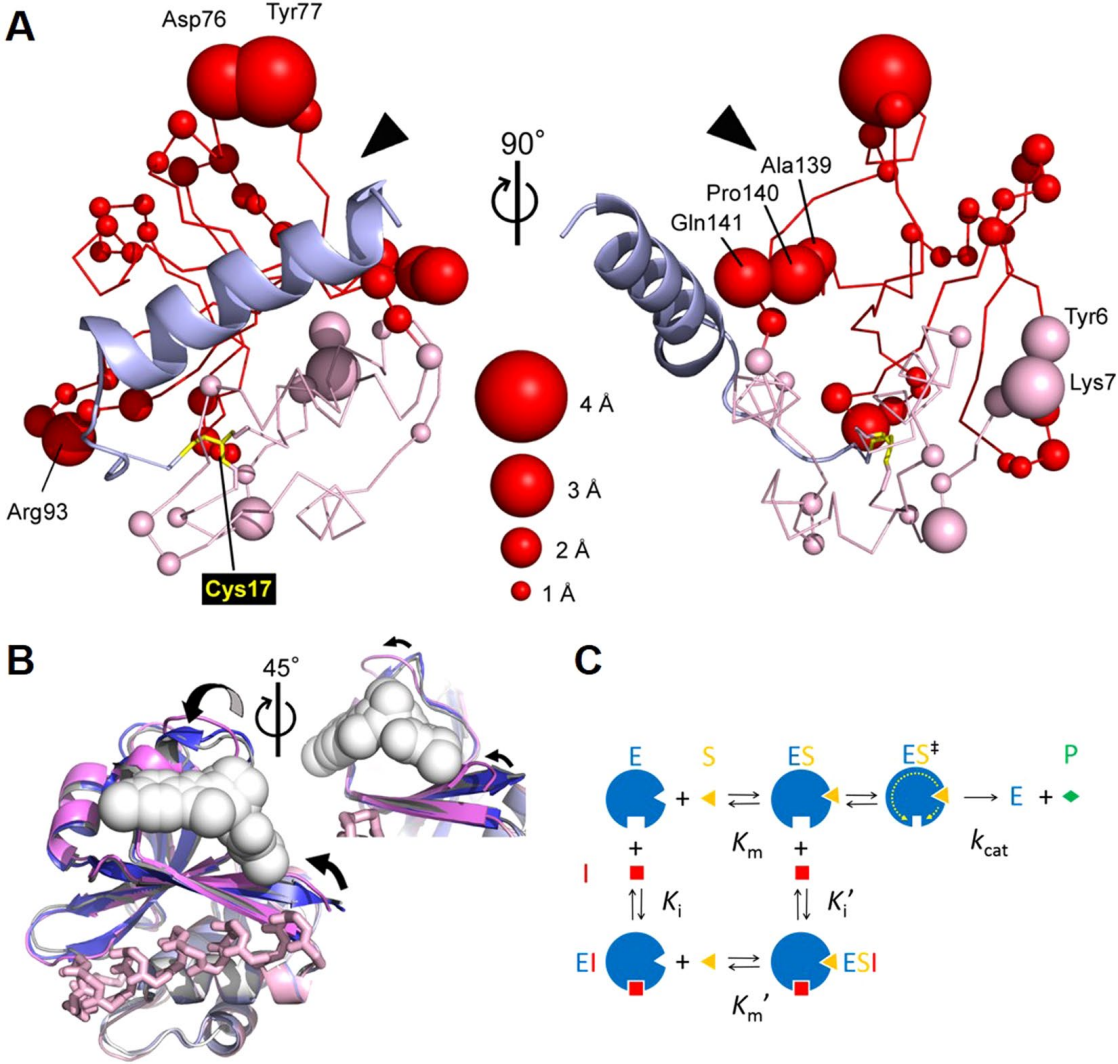
Structural Changes of the Allosteric Site and the Proposed Inhibition Mechanism. (**A**) The acyl-intermediate model of MuPEP1-MuComC, in which the thioester bond is formed between Cys17 of PEP and the cleavage site Gly of MuComC (blue), was obtained by molecular dynamics simulation^18^. The positional difference for each C_*α*_ atom between the free MuPEP1 (PDB ID code 3K8U) and the acyl-intermediate model was calculated and shown. Residues that showed significant differences (≥1 Å) are indicated as spheres, of which the diameters define relative difference distances. Arrowheads indicate the binding site of Compound 2. The N-terminal (1–62) and the C-terminal (63–150) subdomains are colored in light and deep tones, respectively. Arg93, which shows a large structural shift, is located at the entrance to the narrow cleft of the Gly–Gly binding site. The structural changes around Arg93 upon formation of the acyl-intermediate might be propagated and amplified to cause large structural changes around the remote Compound 2-binding site. (**B**) Superimposition of the overall structures of the tMuPEP1-Compoud 2 complex (gray), the free MuPEP1 (blue), and the acyl-intermediate model of MuPEP1-MuComC^18^ (pink). MuComC and Compound 2 are shown as stick and sphere models, respectively. The distinction between light and deep tones is the same as in (**A**). (**C**) The reaction scheme of MuPEP1 and mechanism for non-competitive inhibition. E, enzyme; S, substrate; P, product; I, inhibitor; *K*_m_, Michaelis constant; *k*_cat_, turnover number; and *K*_i_, inhibition constant. After the substrate binding step, the reaction proceeds via a transition state (ES^‡^), in which the space of a pocket on the protein surface is squeezed (broken arrows). The inhibitor binds to this pocket and inhibits the enzyme-catalyzed reaction by preventing structural changes. In the non-competitive inhibition model, *K*_i_^′^ is supposed to be the same as *K*_i_.

The question now arises how the inhibitor modulates the peptidase activity of PEP by binding to this remote site. We previously proposed the structure of the acyl-intermediate model obtained by molecular dynamics simulation based on the crystal structure of MuPEP1^18^. Interestingly, when the structure of the acyl-intermediate was compared with that of the free MuPEP1, amino acid residues that were shifted most remarkably were those that comprised the binding pocket for Compound 2 (Fig. 4A). The positions of C_*α*_ atoms of Asp76 and Tyr77 in the loop were shifted by 3.7 Å and 4.4 Å, respectively, and those of Ala139, Pro140, and Gln141 in the C-terminal *β*-strand were shifted by 2.0 Å, 2.7 Å, and 2.7 Å, respectively. Consequently, the space of this pocket was significantly constricted in the acyl-intermediate model. Superimposition of the acyl-intermediate model and the tMuPEP1-Compound 2 complex structure showed direct collision between the bound inhibitor and some residues lining the pocket (Fig. 4B). Taken together, the following inhibition mechanism is proposed: After substrate binding, PEP catalysis proceeds *via* an acyl-intermediate-like transition state in which a pocket on the protein surface is compressed. When an inhibitor binds to this pocket, the inhibitor prevents the structural changes and non-competitively inhibits the PEP activity (Fig. 4C). To support this hypothesis, the bulky side chain of an arginine was introduced into this pocket at the position of Ala70 (Fig. 3B), which resulted in the catalytic efficiency decreasing to approximately 1% (the *k*_cat_/*K*_m_ of the wild-PEP was 69 M^−1^s^−1^ and that of the Ala70Arg mutant was 0.73 M^−1^s^−1^). The significant effect of the mutation at this remote site implies the importance of the pocket during the catalysis. Recently, such “secondary” binding sites have been explored as druggable sites in many proteins by fragment-based drug screening^22^.

### Effects of MuPEP1 Inhibitors on *S. pneumoniae* PEP, *S. oralis* PEP, and the Peptidase Domain of *S. pneumoniae* BlpA

To examine whether Compound 1 inhibits PEPs from other streptococcal species, *S. pneumoniae* PEP^15^ and *S. oralis* PEP^17^ were chosen, both of which showed moderate homology with MuPEP1 (57% and 59%, respectively, identity in the amino acid sequence). Compound 1 inhibited *S. pneumoniae* PEP and *S. oralis* PEP, with IC_50_ values of 29 *μ*M and 25 *μ*M, respectively (Supplementary Fig. S3). Additionally, 80% of 85 compounds selected after the second screening were found to efficiently inhibit PEP from *S. pneumoniae* (Supplementary Fig. S5). These results support the idea that all PEPs catalyze the reaction through the same catalytic process described above and that development of an inhibitor that is effective in various species of *Streptococcus* might indeed be possible.

BlpA is a ComA-like ABC-transporter that is responsible for the processing and secretion of a bacteriocin, BlpC^23^. BlpC has the Gly–Gly motif at the cleavage site and the four conserved hydrophobic residues in the N-terminal leader region^17^ (Supplementary Fig. S6). The amino acid residues composing the inhibitor-binding pocket of PEPs were also conserved in the peptidase domain of BlpA (Fig. 3D). Indeed, the peptidase domain of BlpA (67% amino acid identity with MuPEP1) efficiently cleaved the fluorescent-labeled substrate tCComC-AFC (Supplementary Fig. S1), and Compound 1 inhibited the peptidase domain of BlpA, with an IC_50_ value of 16 *μ*M.

These results indicate the possibility that Compound 1 inhibits a wide variety of the peptidase domains of bacterial ComA-like ABC transporters, including those of *Streptococcus* commensal species, some of which may function in the maintenance of the commensal flora. Thus, it should be noted that this inhibitor might disturb the beneficial effect of the nasopharyngeal commensals when used as a drug.

## Conclusions

In this study, we focused on PEP as the target of potential inhibitors of the *Streptococcus* quorum-sensing pathway, established a reliable high-throughput screening system using a synthetic substrate for PEP, and successfully obtained a compound that was found to allosterically inhibit PEP and suppress the quorum-sensing pathway, which eventually led to attenuated biofilm formation. Compound 1, albeit with a modest EC_50_ of 5 *μ*M, could be a candidate molecule for further drug development, and the present results could serve as proof-of-principle for the attempt to develop small-molecule inhibitors of the quorum-sensing system, which can affect multiple clinical conditions but have negligible antimicrobial activity.

## Methods

### Materials

The fluorogenic peptide tCComC-AFC, Ala-Gln-Phe-Pro-Val-Leu-Asn-Glu-Lys-Glu-Leu-Lys-Glu-Val-Leu-Gly-Gly-AFC, was purchased from Scrum (Tokyo, Japan). The chemical compound library consisting of 164,514 compounds was supplied from Drug Discovery Initiative, University of Tokyo (Tokyo, Japan). The chemically defined medium (CDM) contains 58 mM K_2_HPO_4_, 15 mM KH_2_PO_4_, 10 mM (NH_4_)_2_SO_4_, 35 mM NaCl, 2 mM MgSO_4_, 4 mM L-glutamate, 1 mM L-arginine monohydrochloride, 1.3 mM L-cysteine, 0.1 mM L-tryptophan, 0.2% casamino acids (Nippon Pharmaceutical, Tokyo, Japan), 44 mM glucose, and 1 × Kao and Michayluk vitamin solution (Sigma-Aldrich, St. Louis, MO). The *Streptococcus*–*E. coli* shuttle vector pSET2^24^ and its host strain, *E. coli* MC1061, were obtained from National Agriculture and Food Research Organization and National BioResource Project (NIG, Japan), respectively.

### Plasmid Constructions for Expression of the Peptidase Domains

The expression plasmids for tMuPEP1 were constructed by using the PrimeSTAR mutagenesis method (Takara, Otsu, Japan) using the MuPEP1 expression plasmid, pSMuP1, as the template^17^. The primers used were 5′-ACATATGTATAAGCTAGTACCTCAGATTGATAC-3′ (forward) and 5′-AGCTTATACATATGTATATCTCCTTCTTAAAGT-3′ (reverse), respectively. For the Ala70Arg tMuPEP1, the single mutation was introduced into the expression plasmid of tMuPEP1 by using primers 5′-ATCAAGCGTGATATGACGCTTTTTGATTATAAT-3′ (forward) and 5′-CATATCACGCTTGATAGAGCGTGTTTCAAAGCC-3′ (reverse). The nucleotide sequences of the entire coding regions were verified.

For the peptidase domain of *S. pneumoniae* G54 BlpA, the dsDNA corresponding to the coding region (GenBank CP001015, bases 475628–476077) with *Nde*I and *Sal*I sites on the 5′ and 3′ terminal ends, respectively, was chemically synthesized and cloned into pEX-A2J1 (Eurofins Genomics K. K. (Tokyo, Japan)). A His_6_-tag sequence was attached to the C-terminal end for the convenience of the purification. The coding DNA was digested with *Nde*I and *Sal*I and ligated into pET21-b to generate the pSPB1.

The expression plasmids for *S. pneumoniae* PEP and *S. oralis* PEP have been reported previously^15, 17^.

### Protein Expression and Purification

For the high-throughput screening and enzyme assay, MuPEP1, *S. pneumoniae* PEP, and *S. oralis* PEP were heterologously expressed in *E. coli* BL21 (DE3) pLysS and purified as previously described^14, 16^. Briefly, *E. coli* cells, carrying each expression plasmid, were grown and induced by 0.2 mM isopropyl-*β*-D-thiogalactopyranoside for 2 h at 37 °C for MuPEP1 or for 5 h at 30 °C for *S. pneumoniae* PEP. The PEPs were purified with His·Bind resin (Novagen, Madison, WI) and dialyzed at 4 °C against a buffer containing 20 mM Tris–HCl, 200 mM ammonium sulfate, and 0.1 mM DTT, pH 7.0.

For crystallization, tMuPEP1 was heterologously expressed in *E. coli* Rosetta™ 2 (DE3) pLysS. *E. coli* cells carrying the expression plasmid were grown and induced by 0.2 mM isopropyl-*β*-D-thiogalactopyranoside for 3 h at 37 °C. The tMutPEP1 was purified by using the same procedure as that for MuPEP1. The purified protein was dialyzed at 4 °C against a buffer containing 70 mM sodium phosphate and 0.1 mM DTT, pH 7.0.

The catalytic activity of tMuPEP1 was identical to that of MuPEP1.

The Ala70Arg tMuPEP1 and the peptidase domain of *S. pneumoniae* BlpA were expressed and purified by using the same method.

### High-throughput Screening

To establish a rapid and robust enzyme assay, we prepared the fluorogenic peptide tCComC-AFC, which corresponds to the N-terminal leader region (−17 to −1) of ComC from *S. cristatus* derivatized with 7-amino-4-(trifluoromethyl) coumarin (AFC) at the C-terminal end. This region of ComC is necessary and sufficient for the interaction with PEP^17^. The [S]−*v*/[E] plots of the PEP assay for tCComC-AFC fit well to the typical Michaelis–Menten curve like those for natural substrates^15, 17^. Thus, this synthetic peptide was used as a functionally relevant substrate. Assays were performed in black flat-bottom 384-well plates (Greinar, 784900) under ambient conditions. All compounds dissolved in DMSO were predispensed on the plate (100 nL/well) with the final concentration of 20 *μ*M, and then aliquots of 5 *μ*L of 0.5 *μ*M MuPEP1 (final concentration) in 50 mM Tris-HCl, 150 mM ammonium sulfate, and 0.02% Triton X-100 were dispensed to each well by using a Multidrop Combi dispenser (Thermo Fisher Scientific, Vantaa, Finland). Reactions were initiated by adding aliquots of 5 *μ*L/well of 10 *μ*M tCComC-AFC (final concentration) in 50 mM Tris-HCl, 150 mM ammonium sulfate, 4% DMSO, and 0.02% Triton X-100 by using the dispenser. These reaction mixtures contained DMSO at a 3% final concentration. Each 384-well plate contained 16 negative control wells (lacking MuPEP1) and 16 positive control wells (100 nL of DMSO replacing 100 nL of compound in DMSO). After 3-h incubation, the fluorescence intensity of the released AFC was measured by using a microplate reader, PHERAstar (BMG Labtech, Offenburg, Germany), with excitation at 380 nm and emission at 490 nm. An inhibition rate for each compound was calculated as follows:

1 $${\rm{Inhibition}}( \% )=100\times (1-({{\rm{FL}}}_{{\rm{c}}}-{{\rm{FL}}}_{{\rm{n}}})/({{\rm{FL}}}_{{\rm{p}}}-{{\rm{FL}}}_{{\rm{n}}}))$$

where FL_c_ is the fluorescence intensity of the well containing the tested compound, FL_p_ is the fluorescence intensity of the well containing the positive control, and FL_n_ is the fluorescence intensity of the well containing the negative control.

### High-performance Liquid Chromatography Assay

Fifteen microliters of 3.3 mM of phosphoric acid was added to 8 *μ*L of the reaction mixtures from the screening. Then, 7.5 *μ*L of those mixtures were loaded onto a YMC Triart C_18_ reversed-phase column (3.0 × 50 mm, 3-*μ*m particle diameter) (YMC, Kyoto, Japan) connected to a LC2000 high-performance liquid chromatography system equipped with an autosampler (Jasco, Tokyo, Japan), and AFC was separated from the unreacted tCComC-AFC by using a mobile phase consisting of 55% of acetonitrile in water with 0.45% formic acid over 2.5 min at a flow rate of 0.5 mL/min under ambient temperature. The fluorescence was detected by using excitation at 360 nm and emission at 470 nm. An inhibition rate for each compound was calculated as follows:

2 $${\rm{Inhibition}}( \% )=100\times (1-{{\rm{FL}}}_{{\rm{c}}}/{{\rm{FL}}}_{{\rm{p}}})$$

where FL_c_ is the fluorescence intensity of the well containing the tested compound, and FL_p_ is the fluorescence intensity of the well containing the positive control.

### Determination of IC_50_ Value in the Second Screening

Dose-dependent inhibitory activities of compounds against MuPEP1 were examined in quadruplicate. Assays were performed under the same conditions used in the first screening except with various concentrations of compound (0.2, 0.6, 2, 6, and 20 *μ*M). An IC_50_ value for each compound was calculated as follows:

3 $$I{C}_{50}=Hig{h}_{conc}{(\frac{Hig{h}_{conc}}{Lo{w}_{conc}})}^{\frac{50-Hig{h}_{inh( \% )}}{Hig{h}_{inh( \% )}-Lo{w}_{inh( \% )}}}$$

where Low_inh(%)_ is the inhibition (%) directly below 50% inhibition, High_inh(%)_ is the inhibition (%) directly above 50% inhibition, Low_conc_ is the corresponding concentration of tested compound directly below 50% inhibition, and High_conc_ is the corresponding concentration of tested compound directly above 50% inhibition.

### Biofilm Formation and Bacterial Growth Assay

Overnight culture of *S. mutans* strain UA159 (ATCC 700610) in BHI medium was inoculated into 200 *μ*L of CDM per well (3.0 × 10^6^ CFU) of the flat-bottomed 96-well microtiter plate (Corning, 3595). After 18 h of standing incubation at 37 °C in 5% CO_2_ under anaerobic condition in an AnaeroPack system (Mitsubishi Gas Chemical, Tokyo, Japan), the cultured medium was removed, and adherent bacteria were stained with 200 *μ*L of 0.005% crystal violet for 30 min. The wells were washed twice with 300 *μ*L of distilled water and then air dried. The dye was extracted into 200 *μ*L of 60% ethanol, and the biofilm mass was estimated by using a microplate reader (Bio-Rad, Model 680XR) (Bio-Rad, Hercules, CA) to measure the absorbance at 595 nm. The bacterial growth was determined by measuring the turbidity (absorbance at 595 nm) of parallel wells.

For the count of viable cells shown in Fig. 2B, part of the culture was serially diluted and spread onto the BHI plate, and the plates were incubated overnight at 37 °C in 5% CO_2_ under anaerobic condition.

### Transformation Assay

Four microliters of the overnight culture of *S. mutans* in BHI medium was inoculated to 0.4 mL of CDM in the absence or presence of 10 *µ*M Compound 1. After 7.5 h incubation at 37 °C in 5% CO_2_ under anaerobic condition in the AnaeroPack system, 1 *μ*g of plasmid pSET2 was added to the culture. After additional 3-h growth, the cultures were chilled on ice, and serial dilutions of the cultures were plated onto BHI agar plates (for enumeration of total CFU) and onto BHI agar plates containing 1 mg/mL spectinomycin (for enumeration of transformants). Transformation efficiency was determined after 24–48 h incubation at 37 °C in 5% CO_2_ under anaerobic condition.

### Real-time Quantitative RT-PCR

Six microliters of the overnight culture of *S. mutans* in BHI medium was inoculated into 0.4 mL of CDM. After 17 h of incubation at 37 °C in 5% CO_2_ under anaerobic condition in an AnaeroPack system, cells were collected by centrifugation and treated with labiase (Cosmo Bio, Tokyo, Japan), and total RNA was extracted and purified by using a PureLink^™^ RNA Mini kit (Ambion, Austin, TX). Purified RNA was reverse transcribed to cDNA by using SuperScript^®^ VILO™ cDNA Synthesis kit. Quantitative real-time PCR was performed by using a Power SYBR^®^ Green PCR Master Mix (Applied Biosystems, Foster City, CA) on a StepOnePlus Real–Time PCR system (Applied Biosystems). The gene expression levels were calculated by using the ΔΔ threshold cycle (ΔΔ*C*_T_) method^25^. The 16S rRNA gene of *S. mutans* was used as the housekeeping reference. The oligonucleotides to amplify each gene were as follows: 5′-GCTTACCAAGGCGACGATACA-3′ and 5′-GCGTTGCTCGGTCAGACTTTC-3′ for 16S rRNA, 5′-GGACAGCCAAACACTTTCAACTG-3′ and 5′-TTGCCGCGACTGCTTCTC-3′ for *nlmA* (SMU. 150), and 5′-ATGGAATTGTGCAGCAGGTATTG-3′ and 5′-CGCCTGCAGTAGCCATATAAC-3′ for *nlmC* (SMU. 1914C). All experiments using commercially available kits were performed according to the manufacturer’s instructions.

### Kinetic Analysis

The PEP activity was assayed in a 110-*μ*L reaction mixture containing 50 mM Tris-HCl, 150 mM ammonium sulfate, 0.02% Triton X-100, and various concentrations of the substrate. The reaction was started by adding the PEP solution to a final concentration of 0.25–2.0 *μ*M. The reaction was performed at 25 °C, 50 *μ*L of the reaction mixture was transferred to a white 96-well microtiter plate (Thermo Scientific 236105), and the fluorescence intensity was immediately measured by using a Promega GloMax^®^-Multi plus Detection System (Promega, Madison, WI) with excitation at 405 nm and emission at 495–505 nm.

### Structure Determination and Analysis

tMuPEP1 was crystallized by using the sitting drop vapor diffusion method. The 16.4-mg/mL protein solution was mixed with an equal volume of 100 mM Bis-Tris (pH 5.5) containing 200 mM ammonium sulfate and 25% (w/v) polyethylene glycol 3,350. The drop was equilibrated against 800 *μ*L of the reservoir solution at 20 °C. The obtained crystal was soaked in a 2-*μ*L drop of the buffer containing 1 mM Compound 2, 50 mM Bis-Tris (pH 5.5), 100 mM ammonium sulfate, 12.5% (w/v) polyethylene glycol 3,350, and 1% DMSO. After incubation for 1 h at room temperature, the crystal was cryo-cooled without an additive cryoprotectant at −173 °C. The diffraction data at 1.0000 Å wavelength were collected by using a MX225HE detector (Rayonix, Evanston, IL) at SPring-8 BL38B1 (Hyogo, Japan). The diffraction data were processed and scaled by using the HKL2000 program package^26^. The structure was solved by molecular replacement using the structure of MuPEP1 (3K8U) as the search model. The program MOLREP^27^ in CCP4 suite was used for the rotation and translation searches. The model was refined by using the REFMAC5^28^ in CCP4 suite, COOT^29^, and PHENIX^30^ programs. The stereochemistry of the structure was checked by using the program PROCHECK^31^ in CCP4 suite. Although two PEP molecules were found to form a dimer in the crystal, this dimer was estimated to be unstable and non-physiological according to the Protein Interfaces, Surface and Assemblies (PISA) service^32^. No electron density was visible for the N-terminal methionine residues of both subunits and a large part of the C-terminal residues (142–150 of the molecule A and 141–150 of the molecule B), including the His-tag. In molecule B, 13 residues (26–38) were also invisible. In the structure of the tMuPEP1-Compound 2 complex, Compound 2 was bound only in the molecule B. Further details of the crystallographic data and statistics are described in Supplementary Table S1.

## Electronic supplementary material

Supplementary Information
