# Supplementary material for: High-throughput Screening of Small Molecule Inhibitors of the Streptococcus Quorum-sensing Signal Pathway
Source: Sci Rep. 2017 Jun 22;7:4029. doi: 10.1038/s41598-017-03567-2 (PMC5481443; doi:10.1038/s41598-017-03567-2)
Supplement: Supplementary file 1 — Supplementary Information [file 41598_2017_3567_MOESM1_ESM.pdf]

Supplementary Information

High-throughput Screening of Small Molecule Inhibitors of the *Streptococcus* Quorum-sensing Signal Pathway

Seiji Ishii, Kenji Fukui, Satoshi Yokoshima, Kazuo Kumagai, Youko Beniyama, Tetsuya Kodama, Tohru Fukuyama, Takayoshi Okabe, Tetsuo Nagano, Hirotatsu Kojima, Takato Yano

Supplementary Table S1. Data collection and refinement statistics.

**Data collection**	**Complex**	**Free**
Beamline	SPring-8 BL38B1	SPring-8 BL38B1
Detector	Rayonix MX225HE	Rayonix MX225HE
Wavelength (Å)	1.0000	1.0000
Space group	$P2_12_12_1$	$P2_12_12_1$
Cell dimensions		
a, *b*, *c* (Å)	46.8, 59.8, 123	46.8, 59.8, 123
α, β, γ (∘)	90.0, 90.0, 90.0	90.0, 90.0, 90.0
Resolution (Å)	50.0–3.10 (3.21–3.10) [a]	50.0–2.80 (2.85–2.80)
R_{merge} (%) [b]	9.3 (17.7)	6.9 (55.7)
$I/\sigma I$	26.8 (17.5)	29.1 (3.83)
Completeness (%)	99.0 (100)	99.9 (100)
Redundancy	6.3 (5.9)	6.8 (6.9)
**Refinement**		
Resolution (Å)	30.8–3.10	31.55–3.10
No. of reflections	6566	6597
R_{work}/R_{free}	0.247/0.285	0.237/0.275
No. of atoms		
Protein	2042	2040
Ligand/Ion	30	14
Water	8	1
B-factor		
Protein	68.9	76.6
Ligand/Ion	70.0	100
Water	37.0	91.3
r.m.s. deviations [c]		
Bond lengths (Å)	0.014	0.005
Bond angles (degrees)	1.61	0.97
Ramachandran plot		
Most favored (%)	85	92
Allowed (%)	13	6.4
Disallowed (%)	2.3	1.2
Protein Data Bank Code	5XE9	5XE8

[a] Values in parentheses are for the highest resolution shell.
[b] $R_{merge} = \sum_{hkl} \sum_i |Ii(hkl) - \langle I(hkl) \rangle| / \sum_{hkl} \sum_i Ii(hkl)$, where *Ii*(*hkl*) is the observed intensity of the *i*th measurement of reflection *hkl*, and ⟨*I*(*hkl*)⟩ is the mean intensity of reflection *hkl* calculated after scaling.
[c] Root mean square deviations.

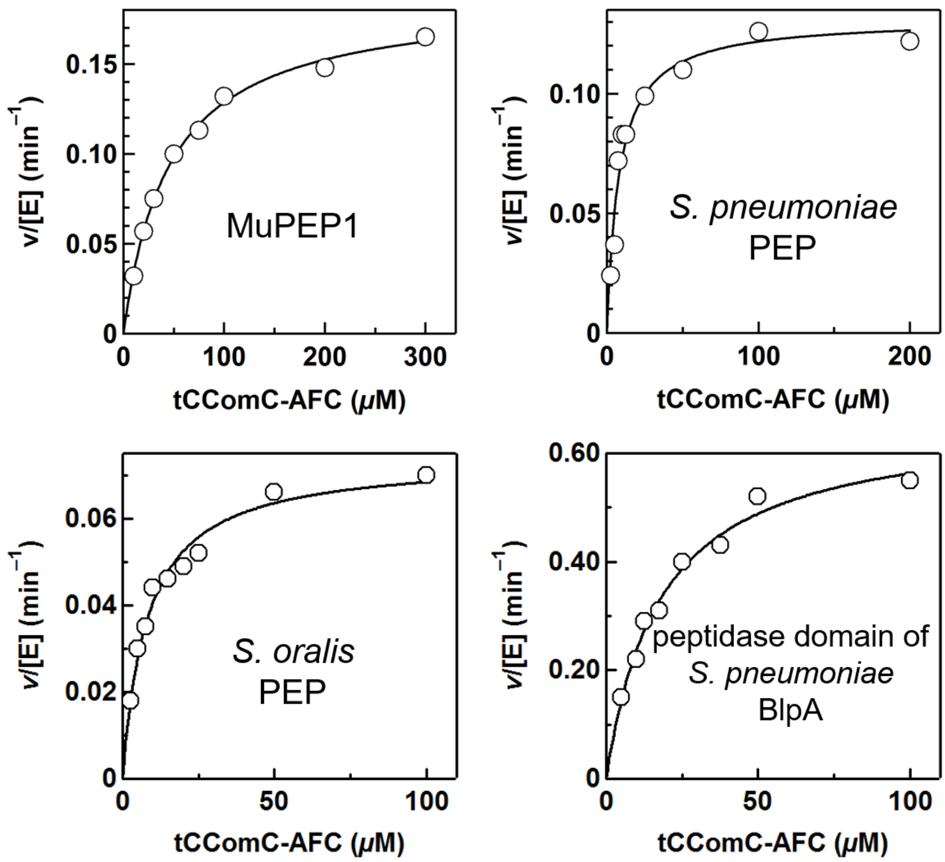

Supplementary Figure S1: *v*/[E] *versus* substrate concentration plots for the PEP assay using tCComC-AFC. The data were fitted with a saturation curve with the parameters of k_{cat} = 0.19 min^{-1} and K_m = 46 μM for MuPEP1, k_{cat} = 0.13 min^{-1} and K_m = 7.9 μM for *S. pneumoniae* PEP, k_{cat} = 0.074 min^{-1} and K_m = 8.2 μM for *S. oralis* PEP, and k_{cat} = 0.66 min^{-1} and K_m = 18 μM for the peptidase domain of *S. pneumoniae* BlpA. Various concentrations of tCComC-AFC were digested in 50 mM Tris-HCl, 150 mM ammonium sulfate, and Triton X-100, pH 7.0, at 25 °C. The concentrations of Triton X-100 were 0.01% for MuPEP1 and 0.02% for *S. pneumoniae* PEP. The concentrations of PEP or the peptidase domain of BlpA were 0.25–2 μM.

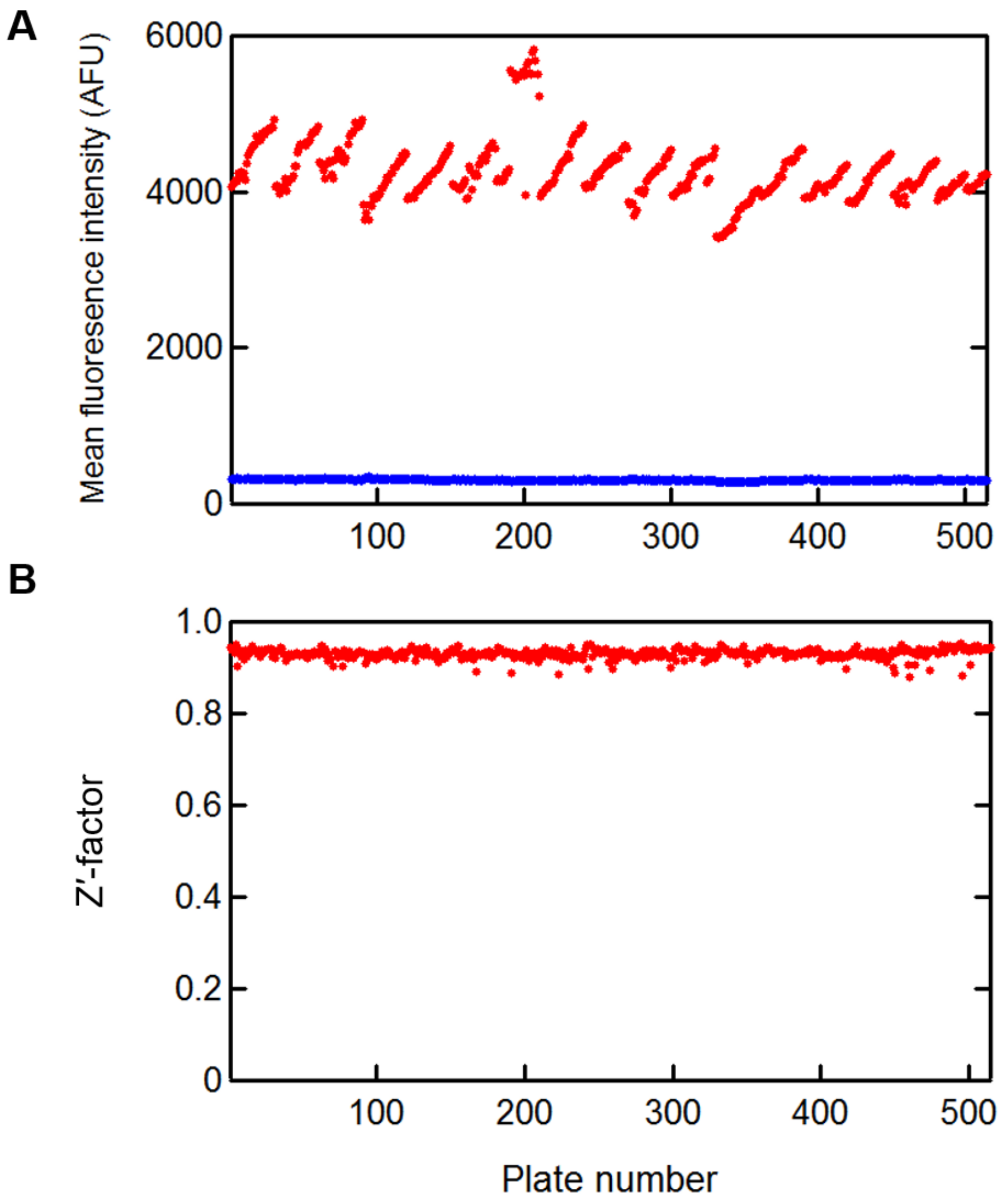

Supplementary Figure S2: High-throughput screening statistics. (A) Mean fluorescence intensities (AFU) of positive control (red) and negative control (blue). (B) The calculated Z′-factor for each plate. A library of 164,514 compounds was screened for the MuPEP1 inhibitory activity in 384-well plates with 16 positive-control wells and 16 negative-control wells per plate. The Z′-factor for each plate was calculated as follows: $Z' = 1 - (3 \times SD_p + 3 \times SD_n) / (M_p - M_n)$, where SD_p is the standard deviation of positive-control wells, SD_n is the standard deviation of negative

control wells, M_p is the mean fluorescence intensity of positive-control wells, and M_n is the mean fluorescence intensity of negative-control wells.

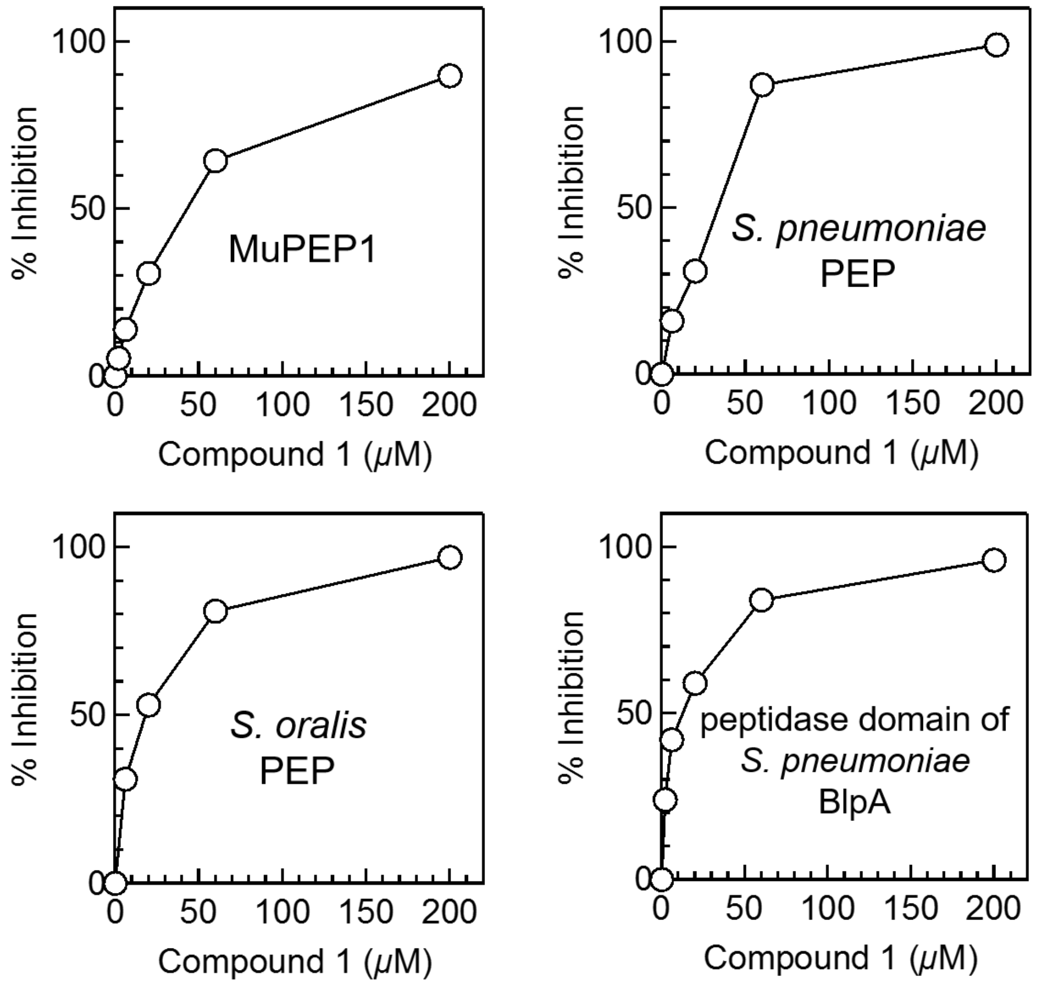

Supplementary Figure S3: Dose-dependent inhibition of PEPs or the peptidase domain of *S. pneumoniae* BlpA with Compound 1. Ten micromolar tCComC-AFC was digested with 0.5 μM PEP or the peptidase domain of BlpA in the presence of various concentrations of Compound 1 in 50 mM Tris-HCl, 150 mM ammonium sulfate, and 0.02% Triton X-100, pH 7.0, under ambient conditions.

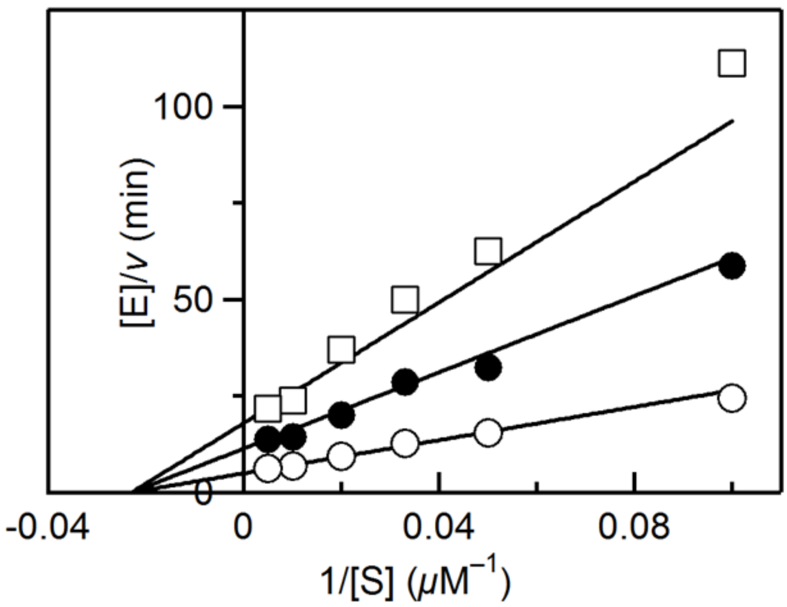

Supplementary Figure S4: Lineweaver–Burk (double-reciprocal) plot of the data of Figure 2D. Various concentrations of tCComC-AFC were digested with 0.5 μM MuPEP1 in 50 mM Tris-HCl, 150 mM ammonium sulfate, and 0.02% Triton X-100, pH 7.0, at 25 **ºC** in the absence (○) or presence (●, 50 μM and □, 100 μM) of Compound 1. The theoretical lines were drawn on the basis of the non-competitive model with the parameters described in the legend under Fig. 2D.

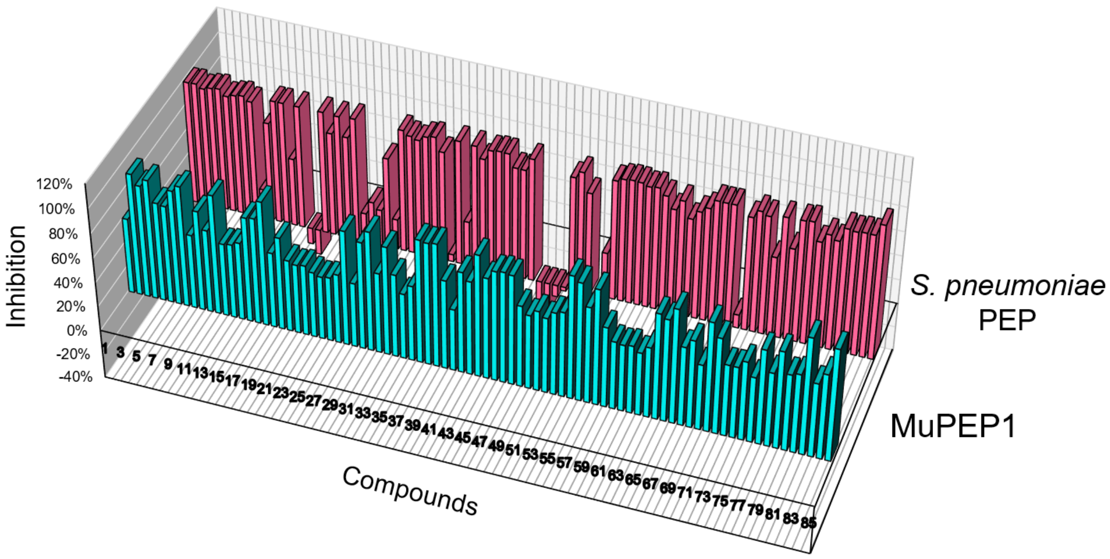

Supplementary Figure S5: Inhibition profiles of 85 compounds selected from the second screening step. Inhibition rates (%) for MuPEP1 (blue) and *S. pneumoniae* PEP (red). Ten micromolar tCComC-AFC was digested in the presence of 20 μM of each compound in 50 mM Tris-HCl, 150 mM ammonium sulfate, and 0.02% Triton X-100, pH 7.0, under ambient conditions. The concentrations of MuPEP1 and *S. pneumoniae* PEP were 0.5 μM and 0.3 μM, respectively.

```
                                     —15  —10  —5  —1
S. pneumoniae ComC MKN-----TVKLEQFVALKEKDLQKIKGGEMRLSKFFRDFILQRKK
S. cristatus ComC  MKNTQKN-FKTIAQFPVLNEKELKEVLGGDLRNIFLKIKFKKK
S. mutans ComC     MKK----TLSLKNDFKEIKTDELEIIIGGSGSLSTFFRLFNRSFTQALGK
S. pneumoniae BlpC MDK-----KQNLTSFQELTTTELNQITGGGLWEDLLYNINRYAHYIT
```

Supplementary Figure S6: Sequence Alignment of ComCs from *S. pneumoniae*, *S. cristatus*, and *S. mutans* and BlpC from *S. pneumoniae*. Highly conserved hydrophobic amino acids and the consensus Gly–Gly are colored red. The arrowhead indicates the cleavage site by PEP.

Supplementary Methods

Synthesis of Compounds

General Remarks: Nuclear magnetic resonance (^{1}H NMR (400 MHz), ^{13}C NMR (100 MHz)) spectra were determined on a ECS400 instrument (JEOL, Tokyo, Japan) unless otherwise noted. Chemical shifts for ^{1}H-NMR are reported in parts per million downfield from tetramethylsilane (δ) as the internal standard, and coupling constants are in Hertz (Hz). The following abbreviations are used for spin multiplicity: s = singlet, d = doublet, t = triplet, q = quartet, m = multiplet, br = broad. Chemical shifts for ^{13}C NMR are reported in ppm relative to the center line of a triplet at 77.0 ppm for deuteriochloroform. Infrared (IR) spectra were recorded on a FT/IR-4100 Fourier Transform Infrared Spectrophotometer (JASCO) and are reported in wavenumbers (cm^{-1}). High-resolution mass spectra were obtained on a JMS-T100LP AccuTOF LC-plus (JEOL) in positive electrospray ionization method using TFANa as the internal standard. Analytical thin-layer chromatography (TLC) was performed on precoated analytical plates (Merck, Darmstadt, Germany), 0.25 mm thick, silica gel 60 F_{254}. Preparative TLC separations were performed on Merck analytical plates (0.25–0.50-mm thick) precoated with silica gel 60 F_{254}. Reagents were commercial grade and used without any purification. ((1*S*,2*R*,4*S*,5*S*)-5-Ethynylquinuclidin-2-yl)methanol (**A**) was prepared from quincoridine according to the procedure reported by Hoffmann and coworkers[1]. Dehydrated tetrahydrofuran was purchased from Kanto Chemicals Co., Inc. (Tokyo, Japan), and purified by using a Glass Contour Solvent System. Dehydrated benzene and *N*,*N*-dimethylformamide were purchased from Kanto Chemicals Co., Inc. and stored over activated MS4A. Dehydrated methanol was also purchased from Kanto Chemicals Co., Inc. and stored over activated MS3A. All reactions sensitive to oxygen or moisture were performed under an argon atmosphere.

((1*S*,2*R*,4*S*,5*S*)-5-Ethynylquinuclidin-2-yl)methyl propylcarbamate (B)

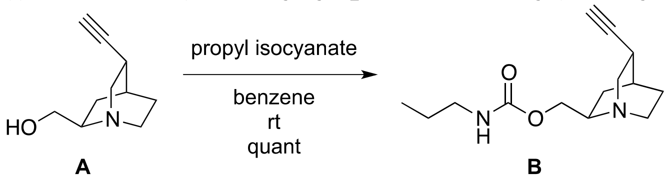

To a solution of **A** (200 mg, 1.2 mmol) in benzene (5.0 mL) was added *n*–propyl isocyanate (110 μL, 1.2 mmol) at room temperature under argon atmosphere, and the resulting mixture was stirred for 16 h. The solution was concentrated in vacuo to afford **B** as a pale-yellow oil. IR (neat, cm^{-1}) 3296, 2940, 2873, 1709, 1538, 1460, 1260, 1145, 1043, 1003, 819; ^{1}H NMR ($CDCl_3$) δ 4.83 (brs, 1H), 4.13–4.04 (m, 2H), 3.13 (dd, *J* = 14, 7.2 Hz, 2H), 3.09–3.05 (m, 2H), 3.02–2.85 (m, 3H), 2.49 (t, *J* = 9.2 Hz, 1H), 2.08 (d, *J* = 2.8 Hz, 1H), 1.93 (d, *J* = 2.8 Hz, 1H), 1.64–1.46 (m, 6H), 0.91 (t, *J* = 7.2 Hz, 3H); ^{13}C NMR ($CDCl_3$) δ 156.5(C), 87.0 (C), 69.3 (CH), 64.1 (CH_2), 54.7 (CH), 48.4 (CH_2), 48.1 (CH_2), 42.6 (CH_2), 28.0 (CH), 27.2 (CH), 25.2 (CH_2), 24.0 (CH_2), 23.0 (CH_2), 11.2 (CH_3); HRMS (ESI+) 251.1752 (calc'd. for $C_{14}H_{23}N_2O_2$ 251.1756).

((1*S*,2*R*,4*S*,5*S*)-5-(Phenylethynyl)quinuclidin-2-yl)methyl propylcarbamate (Compound 1)

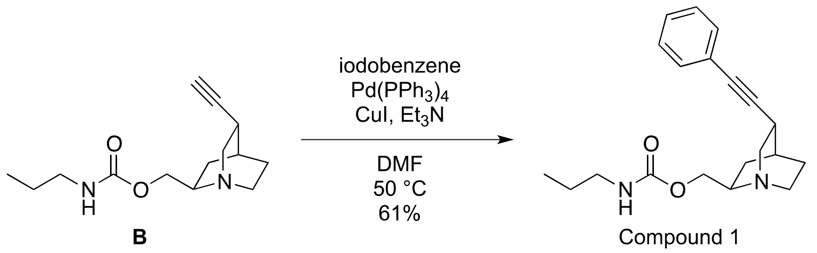

A solution of **B** (50 mg, 0.20 mmol), iodobenzene (20 μL, 0.18 mmol), and Et_3N (50 μL, 0.36 mmol) in DMF (1.0 mL) was degassed by argon bubbling for 15 min. To the solution were added $Pd(PPh_3)_4$ (12 mg, 0.010 mmol) and CuI (5.0 mg, 0.027 mmol), and the resulting mixture was stirred at 50 °C for 2 h. The reaction mixture was concentrated *in vacuo*, and the residue was purified by preparative TLC (6% MeOH in CH_2Cl_2) to afford Compound 1 (40 mg, 0.123 mmol, 61%) as a yellow oil. IR (neat, cm^{-1}) 3323, 2939, 2872, 1713, 1533, 1459, 1259, 1144, 1043, 758; ^1H NMR ($CDCl_3$) δ 7.40 (m, 2H), 7.28 (m, 3H), 4.87 (brs, 1H), 4.18–4.10 (m, 2H), 3.17–3.07 (m, 5H), 2.96–2.88 (m, 2H), 2.71 (dd, *J* = 8.3, 8.3 Hz, 1H), 2.02 (br, 1H), 1.62 (brs, 4H), 1.45 (ddd, *J* = 22.0, 14.6, 7.3 Hz, 2H), 0.90 (t, *J* = 7.3 Hz, 3H); ^{13}C NMR ($CDCl_3$) δ 156.5 (C), 131.6 (CH), 128.2 (CH), 127.7 (CH), 123.5 (C), 92.5 (C), 81.7 (C), 64.2 (CH_2), 54.2 (CH), 48.5 ($2CH_2$), 48.5 (CH_2), 42.7 (CH_2), 29.0 (CH), 27.5 (CH), 25.4 (CH_2), 24.3 (CH_2), 23.1 (CH_2), 11.2 (CH_3); HRMS (ESI+) 327.2081 (calc'd. for $C_{20}H_{27}N_2O_2$ 327.2073).

((1*S*,2*R*,4*S*,5*R*)-5-(3-(4-Methoxyphenyl)-1-methyl-1*H*-pyrazol-5-yl)quinuclidin-2-yl)methyl propylcarbamate (Compound 2)

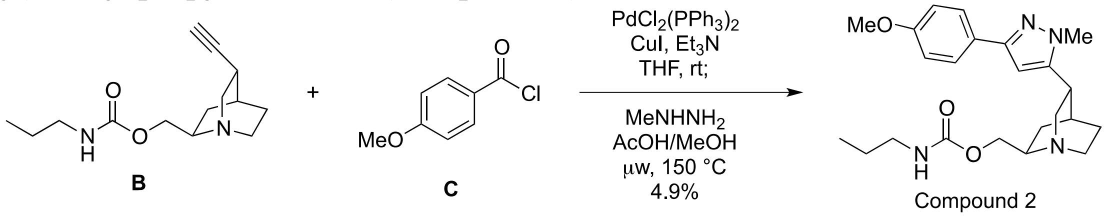

To a solution of $PdCl_2(PPh_3)_2$ (0.8 mg, 1.3 μmol) and CuI (0.4 mg, 2.5 μmol) in degassed THF (0.2 mL) in a microwave tube were added **B** (16 mg, 0.064 mmol), 4-methoxybenzoyl chloride (**C**, 11 mg, 0.064 mmol), and Et_3N (10 μL, 0.068 mmol), and the resulting solution was stirred at room temperature for 17 h. $MeNHNH_2$ (4.0 μL, 0.070 mmol), methanol (0.10 mL), and glacial acetic acid (0.10 mL) were sequentially added to this suspension, and the reaction mixture was heated at 150 °C in the microwave cavity for 10 min. After cooling to room temperature, the solvent was removed under reduced pressure, and the crude products were purified by preparative TLC (CH_2Cl_2/MeOH = 6/1) to afford Compound 2 (2.7 mg, 6.5 μmol, 4.9%). IR (neat, cm^{-1}) 2939, 1707, 1519, 1438, 1248, 1034; ^1H NMR ($CDCl_3$) δ 7.70 (d, *J* = 8.7 Hz, 2H), 6.92 (d, *J* = 8.7 Hz, 2H), 6.34 (s, 1H), 4.90 (brs, 1H), 4.14–4.06 (m, 2H), 3.84 (s, 3H), 3.80 (s, 3H), 3.27 (dd, *J* = 13.7, 10.1 Hz, 1H), 3.15–3.03 (m, 6H), 2.93 (dd, *J* = 9.6, 8.7 Hz, 1H), 2.00 (s, 1H), 1.77 (dd, *J* = 6.0, 6.0 Hz, 2H), 1.56–1.40 (m, 4H), 0.90 (t, *J* = 7.3 Hz, 3H); ^{13}C NMR ($CDCl_3$) δ 159.2 (C), 156.5 (C), 149.9 (C), 145.4 (C), 126.7 (CH), 126.2 (C), 114.0 (CH), 100.5 (CH), 64.1 (CH_2), 55.3 (CH_3), 55.0 (CH), 48.7 (CH_2), 46.9 (CH_2), 42.8 (CH_2), 36.4 (CH_3), 33.5 (CH), 26.8 (CH), 26.5 (CH_2), 23.4 (CH_2), 23.1 (CH_2), 11.2 (CH_3); HRMS (ESI+) 413.2569 (calc'd. for $C_{23}H_{33}N_4O_3$ 413.2553).

Supplementary Reference

1. Schrake, O., Braje, W., Hoffmann, H. M. R. & Wartchow, R. Synthesis of 10,11-didehydro-and 10,11-dihydro-Quincorine and of the Quincoridine analogs: functionalized and enantiopure 1-azabicyclo [2.2.2]-octanes with four stereogenic centers. *Tetrahedron: Asymmetry* **9**, 3717–3722 (1998).
